# Pregnancy and delivery after spine fracture or surgery: A nationwide population-based register study in Finland

**DOI:** 10.1371/journal.pone.0272579

**Published:** 2022-08-05

**Authors:** Matias Vaajala, Ilari Kuitunen, Lauri Nyrhi, Ville Ponkilainen, Maiju Kekki, Tuomas Huttunen, Heikki Mäntymäki, Ville Mattila

**Affiliations:** 1 Faculty of Medicine and Life Sciences, University of Tampere, Tampere, Finland; 2 Department of Pediatrics, Mikkeli Central Hospital, Mikkeli, Finland; 3 Institute of Clinical Medicine and Department of Pediatrics, University of Eastern Finland, Kuopio, Finland; 4 Department of Surgery, Central Finland Central Hospital Nova, Jyväskylä, Finland; 5 Department of Obstetrics and Gynecology, Tampere University Hospital, Tampere, Finland; 6 Center for Child, Adolescent and Maternal Health Research, Faculty of Medicine and Health Technology, Tampere University, Tampere, Finland; 7 Department of Cardiothoracic Anesthesia, Tampere Heart Hospital, Tampere University Hospital, Tampere, Finland; 8 Department of Orthopaedics and Traumatology, Tampere University Hospital, Tampere, Finland; University of Toronto, CANADA

## Abstract

**Background:**

The incidences of spine fractures and fusion surgeries have increased. A few studies have reported an increased rate of caesarean sections (CS) in women who have undergone spine surgery but have not reported on the health of neonates.

**Objective:**

We report the incidence of spine fractures, spine fracture surgeries and fusion surgery for other reasons and the effect of these injuries and procedures on later pregnancy outcomes in Finland.

**Methods:**

Data on all fertile-aged women (1998–2018) who had undergone spine fracture or spine fusion surgery were retrieved from the Care Register for Healthcare and combined with data from the National Medical Birth Register. Women with spine fracture or spine surgery before pregnancy were compared with women without previous spine fracture or surgery. We calculated incidences of spine fracture, spine fracture surgery and fusion surgery for other reasons with 95% confidence intervals (CI). We used multivariable logistic regression to evaluate CS and neonatal health. Results are reported as adjusted odds ratios (AOR).

**Results:**

The main finding of our study was the increasing incidence (156%) of spine fusion surgeries for other reasons in fertile-aged women. A total CS rate (including elective and unplanned CS) in the spine fracture group was 19.7% (AOR 1.26, CI 1.17–1.34), in fusion surgery for other reasons group 25.3% (AOR 1.37, CI 1.30–1.49) and 15.9% in the control group. The rate for neonates requiring intensive care in the spine fracture group was 12.2% (AOR 1.18, CI 1.08–1.29), in fusion surgery for other reasons group 13.6% (AOR 1.12, CI 1.02–1.23) and 10.0% in the control group.

**Conclusions:**

The incidence of fusion surgery for other reasons increased during our study period. The rate of CS was higher in women with preceding spine fracture or fusion surgery. Our results suggest that vaginal delivery after fractures of the spine is both possible and safe for mother and neonate.

## Introduction

Fractures of the spine are typically caused by high-energy trauma and usually occur anatomically at the junction of the thoracic and lumbar spine (Thoracic vertebrae 10–12) [1]. According to the literature, the most common cause of spine fractures in younger patients is falling from height, followed by traffic collisions/accidents and high impact sports. The majority of these high energy spine fractures in younger patients are in the thoracic or lumbar vertebrae [2].

In Finland, the incidence of spine fractures leading to hospitalisation in all patients older than 20 years increased from 57/100 000 person-years in 1998 to 89/100 000 person-years in 2017. Furthermore, a corresponding increase was also observed in the incidence of spine fracture surgeries (from 5.3/100 000 to 8.8/100 000 person-years). Among women, the rate of spine fracture surgeries increased by 147% during the same period [3]. In addition to spine fracture surgery, scoliosis surgery is a more common procedure in younger adults and teenagers. According to von Heideken et al., the annual incidence of scoliosis surgery in Sweden between 2000 and 2013 was estimated to be 12.5/100 000 person-years, with a rapidly increasing trend among women [4].

Moreover, anterior spinal surgery or scoliosis surgery affected the mode of delivery and increased the number of caesarean sections (CS) and led to more frequent preterm deliveries when compared to the population without operation [5–7]. Furthermore, patients who undergo spine surgery have been reported to sustain higher rates of complications related to pregnancy and delivery. A previous small local study reported unchanged delivery rates and neonatal health after surgically treated scoliosis [5].

In Finland, incidences of spine fractures or major surgical spine operations for the whole population have been extensively studied. There is, however, a scarcity of studies on the effects of spine fractures or surgical spine operations on reproductive health, although a few small studies have investigated the effects on delivery and the health of the neonate [5, 7]. This lack of information on the effects of spine fractures and surgeries on the reproductive health of fertile-aged women makes the study of the incidence of spine fractures and surgeries and subsequent pregnancies after spine fracture or surgery of the utmost importance.

The aim of this nationwide register study was therefore to report the incidence of spine fracture, spine fracture surgery and fusion surgery for other reasons in fertile-aged females and to investigate their impact on pregnancies and deliveries.

## Methods

In this nationwide register-based study, we combined data from two national registers–the Care Register for Healthcare and the National Medical Birth Register (MBR). The quality and coverage of both registers is high [8–10]. Both registers are maintained by the Finnish Institute for Health and Welfare. Data on all deliveries and neonates were collected from the MBR, which contains information on all pregnancies, delivery statistics and the perinatal outcomes of births with a birthweight of ≥500 grams or a gestational age of ≥22^+0^ [11]. Data from both registers were then combined by using the pseudonymised identification number of the mother. The study period was from 1998 to 2018.

We differentiated three groups of problems related to the spine–spine fracture, spine fracture surgery and fusion surgery for other reasons. Spine fracture was defined as a hospitalisation period with spine fracture ICD-10 codes. All fertile-aged (15–49 years) women with a spine fracture were included. For each patient, the first spine fracture diagnosis per patient was classified as a separate spine fracture. This was important as the control appointments for the fracture could occur after a long period, and thus make it impossible to identify any new fractures during subsequent hospitalisation periods recorded in the Care Register for Healthcare. Those patients who underwent spine fracture surgery or fusion surgery for other reasons were included based on the operation codes of the Nordic version of the NOMESCO (Nordic Medico-statistical Committee, Finnish version approved by the Finnish Institute for Health and Welfare) classification. Women who underwent spine fusion surgery in a hospitalisation period with a spine fracture diagnosis were identified as fracture surgery patients. The spine fracture diagnosis codes and operation codes included in this study are presented in S1 Table.

**Fig 1:**
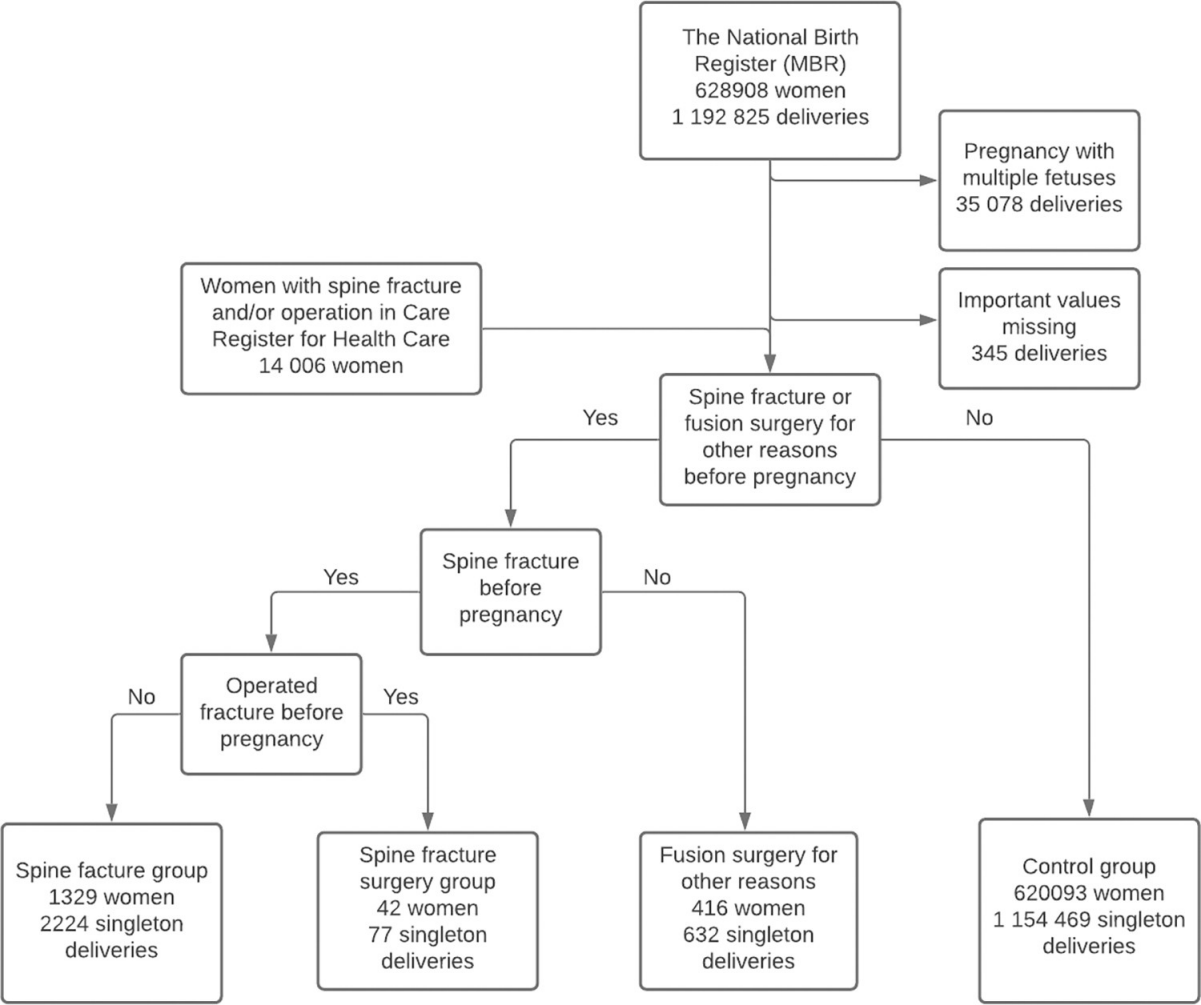
Flowchart of the study population. Data from the MBR were combined with data on the diagnosed spine fractures and spine operations recorded in the Care Register for Healthcare.

Women with a spine fracture prior to delivery formed the fracture group, which was then categorized into operated and non-operated fracture patients. A total of 1371 women with 2301 singleton deliveries were identified in the group of women with previous spine fracture. Of these, 734 women with 1234 deliveries suffered a fracture in lumbar spine. Conservatively treated fracture patients (1329 women with 2224 singleton deliveries) and surgically treated fracture patients (42 women with 77 singleton deliveries) were analysed separately. For clarity, they are presented together as the fracture group in tables, and only remarkable findings have been presented separately. Women with fusion surgery for other reasons included 416 women with 632 singleton deliveries. Of these, 206 women with 309 deliveries had a fusion surgery for other reasons in lumbar spine. The control group consisted of 620 093 women who had 1 154 469 singleton deliveries without a preceding spine fracture, spine fracture surgery or fusion surgery for other reasons (Fig 1). Deliveries with missing information on the mode of delivery were excluded. In this study, each non-elective caesarean section (CS) is considered an unplanned CS. The results of this study are reported according to the STROBE guidelines [12].

### Ethics

Both the National Medical Birth Register (MBR) and the Care Register for Healthcare had the same unique pseudonymised identification number for each patient. The pseudonymisation was made by the Finnish data authority FINDATA, and the authors did not have access to the pseudonymisation key, as it is maintained by FINDATA. In accordance with Finnish legislation, no informed written consent was required because of the retrospective register-based study design and because the patients were not contacted. Permission for the use of this data was granted by FINDATA after evaluation of the study protocol (Permission number: THL/1756/14.02.00/2020).

### Statistics

Continuous variables were interpreted as mean with standard deviation or as median with interquartile range based on distribution of the data. Categorized variables were presented as absolute numbers and percentages. Student’s t-test, Mann-Whitney U-test and Chi squared tests were used for group comparisons. Statistical tests were used to compare separately patient groups (spine fracture and fusion surgery for other reasons group) to control group. P-value under 0.05 was considered statistically significant. The multivariable logistic regression model was used to access the primary outcomes (mode of delivery and neonatal health). The model was used separately for fracture patients and patients with fusion surgeries for other reasons. When using the model, the other group were excluded from the data, as it would otherwise occur as part of the control group and cause distortion in the results. As lumbar spine is anatomically located near the reproductive system and therefore the effects of traumas and surgeries on this area on pregnancy and delivery are of great interest, we analysed fractures and surgeries in lumbar spine separately from thoracical and cervical spine. The need for intensive care for the neonate was used as an indicator for neonatal health in logistic regression analysis. Maternal smoking during pregnancy and maternal diabetes during pregnancy were used as adjusting variables. Odds ratios (OR) and adjusted odds ratios (AOR) with 95 confidence intervals (CI) were calculated for the main outcomes. Crude OR were included in the study because of the unreliability of the maternal diabetes variable in the data. Adjustments were made by choosing the variables for the multivariate model using directed acyclic graphs (DAGs) constructed using the free online software DAGitty (dagitty.net). The variables included in the DAGs were chosen based on known risk factors and the hypothesised causal pathways. The DAGs are presented as a supplementary file (S1 and S2 Figs). Statistical analysis was performed using R version 4.0.3.

## Results

### Spine fractures and spine fracture surgery

**Fig 2:**
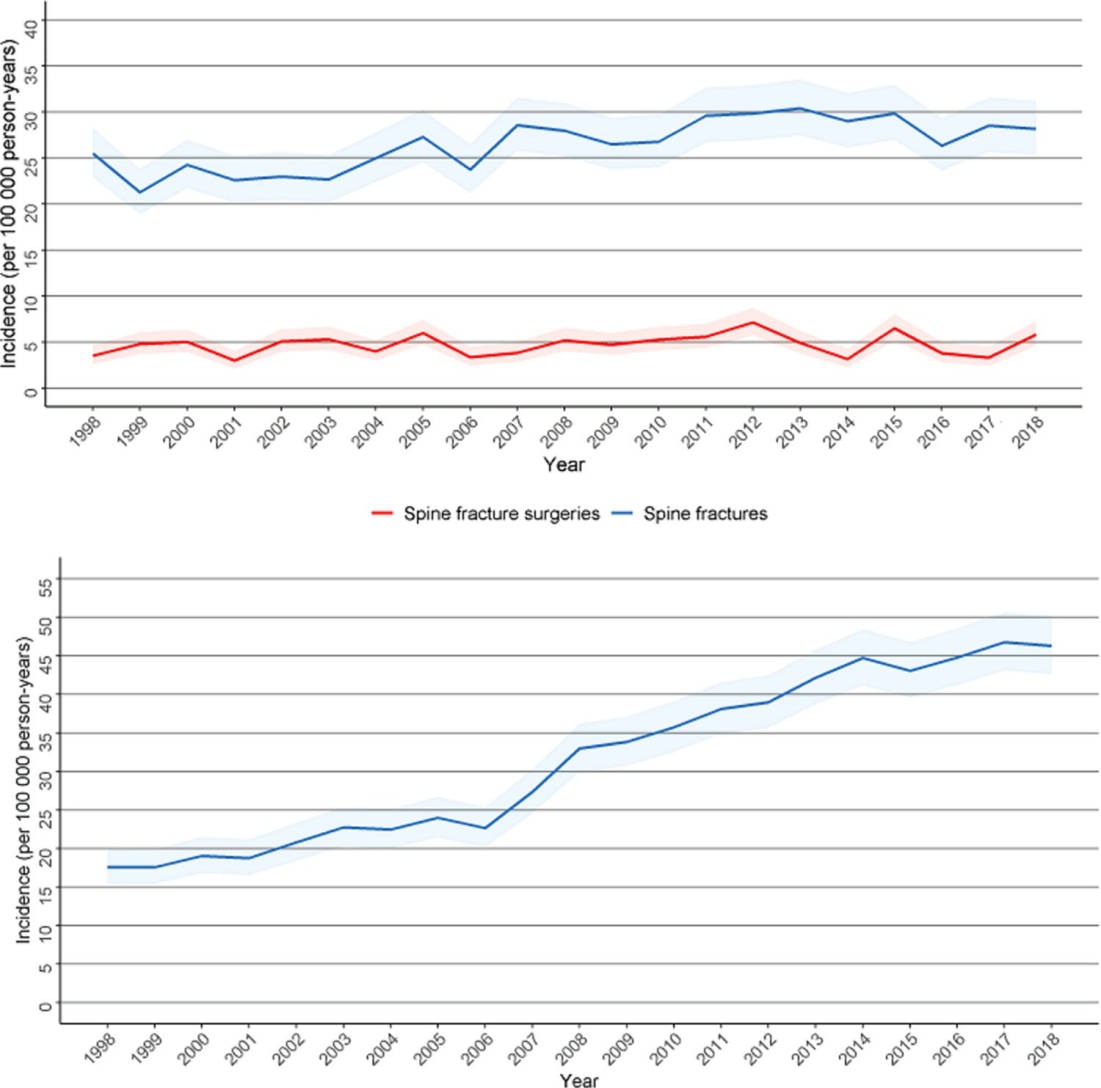
A. Incidence of spine fractures and spine fracture surgeries with 95% confidence intervals among fertile-aged (15–49 years) women during the study period. B. Incidence of fusion surgeries for other reasons with 95% confidence intervals among fertile-aged (15–49 years) women during the study period.

A total of 14 006 women with a spine fracture or who underwent fusion surgery for other reasons were identified from the Care Register for Healthcare. A total of 6374 women had a spine fracture during the study period. The incidence of spine fracture hospitalisation increased slightly during our study period from 24.3 per 100 000 person-years in 1998 to 28.7 per 100 000 person-years in 2018. However, the incidence of spine fracture surgery remained stable during this period (Fig 2A).

### Fusion surgery for other reasons

A total of 416 fusion surgeries for other reasons prior to delivery were identified. The incidence of fusion surgeries for other reasons increased more than twofold from 17.6 per 100 000 person-years to 46.3 per 100 000 person-years (Fig 2B). Anterior fusion of the cervical spine without fixation (38%) and posterior fusion of the lumbar spine with fixation (21%) were the most common operations during the study period, with scoliosis being the most typical reason for these operations. The mean age of the women undergoing the operation NAG 53 (Posterior or lateral fusion of thoracic spine with fixation, more than 3 vertebrae) had a notably lower mean age (19.1) than any other operation (S2 Table).

### Pregnancies and deliveries

Compared to patients without a previous spine fracture or fusion surgery, the rate of nulliparous women was higher in the fracture group (46.1%, p < 0.001) and the fusion surgery group (43.4%, p < 0.001). In the fracture group, a higher percentage of women smoked during pregnancy (27.2%, p < 0.001), whereas only 14.6% of women in the control group smoked (Table 1).

**Table 1:** Background characteristics of women having singleton pregnancies in the patient groups and control group.

	Fracture group		Fusion surgery group		Control group	
Total number	2301		632		1 154 469	
	n	%	n	%	n	%
Age at birth (years, mean SD)	29.4 (4.7)		30.6 (6.2)		29.7 (5.4)	
Nulliparous	1060	46.1	274	43.4	477 595	41.4
Previous C-section	241	10.5	99	15.7	124 013	10.7
Smoking during pregnancy	625	27.2	114	18.0	168 633	14.6

AOR for CS in the spine fracture group was 1.26 (CI 1.17–1.34), and the need for intensive care treatment for the neonate was 1.18 (CI 1.08–1.29). Among women with fusion surgery for other reasons, the AOR for caesarean sections was 1.39 (CI 1.30–1.49), and the need for intensive care treatment for the neonate was 1.12 (CI 1.02–1.23). When comparing only patients with fracture or fusion surgery for other reasons in lumbar spine, the AORs were similar compared to the AORs of the whole spine. (Table 2)

**Table 2:** Univariable and multivariable logistic regression interpreted as odds ratios (OR) with 95% confidence intervals (CI) for the main variables. Both groups were compared with the control group. Also, patients with fracture or surgery in lumbar spine (L) were compared with control group separately. The models were adjusted by maternal smoking and diabetes during pregnancy.

	Caesarean section (fracture group)	Neonatal intensive care (fracture group)	Caesarean section (Fusion surgery group)	Neonatal intensive care (Fusion surgery group)
	OR (95% CI)	OR (95% CI)	OR (95% CI)	OR (95% CI)
Univariable	1.27 (1.17–1.36)	1.23 (1.12–1.35)	1.38 (1.28–1.48)	1.14 (1.04–01.25)
Adjusted	1.26 (1.17–1.34)	1.18 (1.08–1.29)	1.39 (1.30–1.49)	1.12 (1.02–01.23)
Univariable (L)*	1.35 (1.22–1.48)	1.27 (1.13–1.44)	1.46 (1.30–1.65)	1.27 (1.09–1.48)
Adjusted (L)	1.34 (1.21–1.48)	1.23 (1.09–1.39)	1.49 (1.32–1.68)	1.26 (1.08–1.16)

* L meaning only patients with fractures or fusion surgery for other reasons in the lumbar spine.

Patients with spine fracture or fusion surgery without fracture had a slightly higher rate of elective CS when compared to control group (9.5% and 13.1% vs 6.6%, p < 0.001 for both). After excluding elective CS, unplanned CS was on the same level in the fracture group (^11^.3%, p = 0.032) and higher in the fusion surgery group (14.0%, p < 0.001), when compared to the control group (9.9%). In the same analysis, including only primipara and women without previous CS, the rates of unplanned CS were 15.5% (p < 0.001) in the fracture group, 17.2% (p < 0.001) in the fusion surgery for other reasons group and 14.2% in the control group. Epidural and spinal anaesthesia were more common among patients with fracture or fusion surgery when compared to control group (p < 0.001 for both) (Table 3). In addition, pudendal and paracervical analgesia were slightly more common in these groups (p < 0.001). Labour analgesia showed a larger proportional increase in the fracture patients and fusion surgery patients groups when compared to the control group (p < 0.001). (Table 3).

**Table 3:** Proportions of obstetric variables in attempted vaginal deliveries (without elective CS) of the fracture patient groups and the control group. Elective CS was the intended mode of delivery in 218 (9.5%) of all deliveries in fracture group, 83 (13.1%) in fusion surgery for other reasons group and 76 478 (6.6%) in control group.

	Fracture group		Fusion surgery group		Control group	
Total number (without elective CS)	2083		549		1 077 991	
	n	%	n	%	n	%
Mode of delivery						
spontaneous vaginal delivery	1630	78.3	411	74.9	873 485	81.0
Instrumental vaginal delivery	217	10.4	61	11.1	97 666	9.1
unplanned CS	236	11.3	77	14.0	106 844	9.9
Labour analgesia						
epidural	1000	48.0	257	46.8	469 166	43.5
spinal	342	16.4	79	14.4	122 797	11.4
spinal + epidural	58	2.8	14	2.6	13 600	1.3
paracervical	414	19.9	128	23.3	188 203	17.5
pudendal	192	9.2	48	8.7	67 331	6.2

### Neonatal health

Perinatal mortality or problems related to the health of the neonate (1-minute Apgar under 6, delivery related asphyxia, need for phototherapy) were not more common in the fracture group or the fusion surgery for other reasons group. However, a higher proportion of neonates born to women in the spine fracture and fusion surgery for other reasons groups needed intensive care compared to the control group (12.2% and 13.6 vs 10.0%, p < 0.001). (Table 4).

**Table 4:** Perinatal characteristics and outcomes in the patient groups and the control group.

	Fracture group		Fusion surgery group		Control group	
Total number	2301		632		1154469	
	n	%	n	%	n	%
LBW* < 2500g	80	3.5	24	3.8	34 402	3.0
Preterm < 37 + 0 gestational weeks	115	5.0	46	7.3	53 019	4.6
Perinatal mortality**	9	0.4	5	0.8	6158	0.5
1 minute Apgar score ≤ 6	333	14.5	85	13.4	157 131	13.6
Neonatal intensive-care unit	281	12.2	86	13.6	115 558	10.0
Discharged from hospital during the first week	2143	93.1	589	93.2	1 084 983	94.0

*LBW = low birthweight.

**perinatal mortality includes still births and deaths before the age of seven days

### Comment

The main finding of our study was the increasing incidence (156%) of spine fusion surgeries for other reasons in fertile-aged women. The incidence of spine fractures, however, remained nearly unchanged during the study period. Moreover, women with spine fracture or spine fusion surgery for other reasons had higher rates of elective and unplanned CS, but neonatal health was not importantly impaired in either group.

In a recent study on the incidence of spine fracture and spine fusion surgeries in all patients older than 20 years in Finland (1998–2017), the increase was evaluated to be approximately 65% [3]. When compared to this finding, the proportional increase in elective fusion surgery in fertile-aged women was higher in the present study. The exact reason for this increase is unknown, but the rapidly increasing incidence of scoliosis [4] might be one probable explanation for this finding. In addition, based on our results the incidence of spine fracture surgery remained stable in fertile-aged women, which possibly indicates that the increase observed in previous study is mostly due to increase in the older age groups. The previous study also reported that the increase in the whole population was highest in patients over 60-years of age (400%) [3].

Elective and unplanned CS were more common in the fracture and spine fusion surgery groups. In addition, the risk for elective and unplanned CS among patients with fracture or fusion surgery in lumbar spine increased slightly. However, vaginal delivery was possible in most cases. In addition, the need for intensive care for the neonates was little higher in fracture and fusion surgery for other reasons group, but the clinical importance of this remains unclear. Adjusting the models with smoking status and maternal diabetes decreased the AORs for caesarean section and impaired health of neonate, meaning that these have most likely effect on these outcomes in fracture group and fusion surgery for other reasons group, but aren’t an explanation alone. Elective CS rate was two times higher in the spine fusion surgery for other reasons group. This increase is notable because in Finland the indication for CS is always considered carefully between patient and physician. The combined elective and unplanned CS rate in Finland is reported to be 16% [13]. In our study, however, the rate of CS in spine fracture and spine fusion surgery patients is lower than in most Western countries [14]. In a study in the United States, the incidence of elective CS after spine surgery was reported to be 37% [7], which is three times higher than the incidence in our results. These results raise questions about the reasons behind the higher rate of elective CS in patients with spine fracture or fusion surgery, as we did not observe neonatal health to be importantly impaired. Although CS is a fast and relatively safe operation and has played a remarkable role in decreasing mortality in neonates, many disadvantages for the mother and neonate following the operation have been reported. In neonates born by CS, an increased risk for asthma, obesity, and poorer cardiorespiratory health in later life has been reported [15–17]. For mothers, CS has been associated with shorter breastfeeding duration, future subfertility and complications related to future pregnancies [18–21]. Further, these results should be acknowledged by the patient, the obstetrician and the orthopaedic consultant when considering the necessity for elective CS, as vaginal delivery appears still to be safe delivery method. In the present study, the rate of unplanned CS was higher among patients in the fusion surgery for other reasons group. The exact reason for this remains unclear, as no such increase was found in the fracture group. However, there was a higher proportion of nulliparous women in the fusion surgery group, and the women in this group had higher rates of previous CS, which could partly explain the higher rate of elective and unplanned CS. In addition, a slightly higher rate of preterm deliveries in this group could affect the rate of unplanned CS. Another possible explanation might be the awareness of previous spine fusion surgery, which may lower the threshold for the obstetrician to convert the trial of labour to CS. Additionally, some women with a recorded unplanned CS may already have planned an elective CS, but because the labour began early, the planned elective CS was recorded as an unplanned CS. Further, the rate of instrumental vaginal deliveries was higher in the fusion surgery for other reasons group, which could possibly indicate a more challenging vaginal delivery after fusion surgery.

A study with a small number of patients concluded that spinal cord injury did not have a negative effect on the health of neonates [22], but there is little information about the effects of spinal trauma or surgery on neonatal health. Even though women who previously underwent fusion surgery had a higher rate of preterm deliveries, our results suggest that a previous spine fracture or fusion operation does not have a clinically relevant negative effect on the health of the neonate. Indeed, the slightly higher percentages of neonates in need of intensive care might partly be explained by the higher proportional number of CS and preterm deliveries in these groups. However, no clinically important difference was found between the groups in any of the neonatal health indicators.

Interestingly, the rates of labour analgesia provided by anaesthesiologists in the fracture group and the fusion surgery group were remarkably higher when compared to the control group. The literature documenting the management of labour analgesia after spinal surgery or spine fracture is limited to only a few studies with small study populations. According to this quite limited literature, the main problems for the anaesthesiologist are the difficulties associated with performing the procedure. These difficulties include the inability to identify the epidural space, multiple attempts before catheter insertion, vascular trauma, subdural local anaesthetic injection and accidental dural puncture [23]. Based on our results it appears that the rates of labour analgesia was higher in fracture group and fusion surgery for other reasons group. In our study, however, any possible complications during or after anaesthesia remain unknown, as this information is not recorded in the MBR, making it impossible to draw conclusions about the success rate of labour analgesia after spine fracture or surgery. However, current understanding is that epidural analgesia does not raise the risk for CS or instrumental vaginal delivery [24, 25]. One possible explanation for higher rates of labour analgesia in the fracture or fusion surgery group might be the decreased mobility and possible decreased flexibility of the spine, which could create the need for greater pain relief.

The strength of our study is the large nationwide study population with a long study period, enabling the proper analysis of such rare events. The register data we used in our study are routinely collected using structured forms with nationwide instructions, which ensures the good coverage and reduces possible reporting and selection bias [13]. Therefore, the coverage and validity of both registers included in this study are high [10]. The advantage of our study compared to previous studies is the large national research material in a country with uniform delivery-related guidelines and attitudes.

The main limitation of our study is the missing clinical information on fractures and other spine diseases (i.e., radiological findings or pelvimetric examination results). As this information is not recorded to the registers, we could only use ICD-10 coding. Further, the contents of the birth register were updated in 2004 and 2017, and 5-minute Apgar scores, durations of labour stages, body mass index and the chronic disease diagnosis of the mother were only included after 2004. Therefore, these were not analysed in our study. Furthermore, since cases of CS were classified as elective or urgent prior to 2004, we have used the same classifications in the present study instead of the elective, urgent and emergency classifications. Also, the indications behind CS are not registered in the MBR, which means that the indications for elective CS, such as had the patient planned an elective CS or attempted vaginal delivery before unplanned CS, remain unknown.

## Conclusions

Based on the findings of the present study, the incidence of fusion surgery for other reasons had a strongly increasing trend during our study period. The proportion of CS was higher in the spine fracture or fusion surgery for other reasons group when compared to the women (in control group) without spine fracture or operated spine. Moreover, the need for intensive care for neonates born to mothers who underwent spine fracture or fusion surgery for other reasons before pregnancy was little higher, but the clinical importance of this remains unclear. However, our results suggest that vaginal delivery after fractures of the spine is both possible and safe for mother and neonate. These findings could further encourage obstetricians and women with a previous spine operation or fracture to consider the vaginal delivery approach.

## Supporting information

S1 Table Definitions for ICD-10-codes and NOMESCO classification codes for fracture-related and other major spine operations included in this study. (PDF)

S2 Table Frequencies of the spine operations and the mean age of the women at the time of operation in these subgroups during the study period. (PDF)

S1 Fig DAG: Health of neonate as a dependent variable. (TIF)

S2 Fig DAG Risk for caesarean section as a dependent variable. (TIF)

## References

[1] Wood KB, Li W, Lebl DR, Ploumis A. Management of thoracolumbar spine fractures. *The spine journal*: *official journal of the North American Spine Society*. 2014;14(1):145–164. S1529-9430(13)00679-7 [pii] doi: 10.1016/j.spinee.2012.10.041 24332321

[2] Leucht P, Fischer K, Muhr G, Mueller EJ. Epidemiology of traumatic spine fractures. *Injury*. 2009;40(2):166–172. doi: 10.1016/j.injury.2008.06.040 19233356

[3] Ponkilainen VT, Toivonen L, Niemi S, Kannus P, Huttunen TT, Mattila VM. Incidence of Spine Fracture Hospitalization and Surgery in Finland in 1998–2017. *Spine (Phila Pa1976)*. 45(7):459–464. doi: 10.1097/BRS.0000000000003286 31609884

[4] Heideken J von, Iversen MD, Gerdhem P. Rapidly increasing incidence in scoliosis surgery over 14 years in a nationwide sample. *European spine journal*: *official publication of the European Spine Society*, *the European Spinal Deformity Society*, *and the European Section of the Cervical Spine Research Society*. 2018;27(2):286–292. doi: 10.1007/s00586-017-5346-6 29052036

[5] Orvomaa E, Hiilesmaa V, Poussa M, Snellman O, Tallroth K. Pregnancy and delivery in patients operated by the Harrington method for idiopathic scoliosis. *European spine journal*: *official publication of the European Spine Society*, *the European Spinal Deformity Society*, *and the European Section of the Cervical Spine Research Society*. 1997;6(5):304–307. BF01142675 [pii] doi: 10.1007/BF01142675 9391799 PMC3454608

[6] Visscher W, Lonstein JE, Hoffman DA, Mandel JS, Harris 3rd BS. Reproductive outcomes in scoliosis patients. *Spine*. 1988;13(10):1096–1098. doi: 10.1097/00007632-198810000-00006 3206266

[7] Lavelle WF, Demers E, Fuchs A, Carl AL. Pregnancy after anterior spinal surgery: fertility, cesarean-section rate, and the use of neuraxial anesthesia. *The spine journal*: *official journal of the North American Spine Society*. 2009;9(4):271–274. doi: 10.1016/j.spinee.2008.05.007 18619910

[8] Gissler M, Teperi J, Hemminki E, Meriläinen J. Data quality after restructuring a national medical registry. *Scandinavian journal of social medicine*. 1995;23(1):75–80. doi: 10.1177/140349489502300113 7784857

[9] Gissler M, Shelley J. Quality of data on subsequent events in a routine Medical Birth Register. *Medical informatics and the Internet in medicine*. 2002;27(1):33–38. 0UB69R4X61VDGCDA [pii] doi: 10.1080/14639230110119234 12509121

[10] Sund R. Quality of the Finnish Hospital Discharge Register: a systematic review. *Scandinavian Journal of Public Health*. 2012;40(6):505–515. doi: 10.1177/1403494812456637 22899561

[11] Vuori EGM. Perinatal statistics: parturients, deliveries and newborns 2015. National Institute of Health and Welfare; 2016. (Journal Article).

[12] von Elm E, Altman DG, Egger M, et al. The Strengthening the Reporting of Observational Studies in Epidemiology (STROBE) statement: guidelines for reporting observational studies. *JClinEpidemiol*. 61(4):344–349. doi: 10.1016/j.jclinepi.2007.11.008 18313558

[13] THL. Parturients, deliveries and births. 2021 Mar 9 [Cited 2022 Jun 20]. In official website of THL [Internet]. Finland. Available from https://thl.fi/en/web/thlfi-en/statistics/statistics-by-topic/sexual-and-reproductive-health/parturients-deliveries-and-births/perinatal-statistics-parturients-delivers-and-newborns.

[14] Betrán AP, Ye J, Moller AB, Zhang J, Gülmezoglu AM, Torloni MR. The Increasing Trend in Caesarean Section Rates: Global, Regional and National Estimates: 1990–2014. *PloS one*. 2016;11(2):e0148343. doi: 10.1371/journal.pone.0148343 26849801 PMC4743929

[15] Li HT, Zhou YB, Liu JM. The impact of cesarean section on offspring overweight and obesity: a systematic review and meta-analysis. *International journal of obesity (2005)*. 2013;37(7):893–899. doi: 10.1038/ijo.2012.195 23207407

[16] Mueller NT, Zhang M, Hoyo C, Østbye T, Benjamin-Neelon SE. Does cesarean delivery impact infant weight gain and adiposity over the first year of life? *International journal of obesity (2005)*. 2019;43(8):1549–1555. doi: 10.1038/s41366-018-0239-2 30349009 PMC6476694

[17] Ekstrom LD, Ahlqvist VH, Persson M, Magnusson C, Berglind D. The association between birth by cesarean section and adolescent cardiorespiratory fitness in a cohort of 339,451 Swedish males. *Scientific reports*. 2020;10(1):18661–18662. doi: 10.1038/s41598-020-75775-2 33122786 PMC7596509

[18] Hobbs AJ, Mannion CA, McDonald SW, Brockway M, Tough SC. The impact of caesarean section on breastfeeding initiation, duration and difficulties in the first four months postpartum. *BMC pregnancy and childbirth*. 2016;16:90–91. doi: 10.1186/s12884-016-0876-1 27118118 PMC4847344

[19] Ometti VM, Bettinelli G, Candiani M,& Salini. Pelvic bone surgery and natural delivery: absolute and relative contraindications. Lo Scalpello-otodi Educational, 34, 160–164.

[20] Keag OE, Norman JE, Stock SJ. Long-term risks and benefits associated with cesarean delivery for mother, baby, and subsequent pregnancies: Systematic review and meta-analysis. *PLoS medicine*. 2018;15(1):e1002494. doi: 10.1371/journal.pmed.1002494 29360829 PMC5779640

[21] Liu S, Liston RM, Joseph KS, et al. Maternal mortality and severe morbidity associated with low-risk planned cesarean delivery versus planned vaginal delivery at term. *CMAJ*: *Canadian Medical Association journal = journal de l’Association medicale canadienne*. 2007;176(4):455–460. 176/4/455 [pii] doi: 10.1503/cmaj.060870 17296957 PMC1800583

[22] Cross LL, Meythaler JM, Tuel SM, Cross AL. Pregnancy, labor and delivery post spinal cord injury. *Paraplegia*. 1992;30(12):890–902. doi: 10.1038/sc.1992.166 1287543

[23] Kuczkowski KM. Labor analgesia for the parturient with prior spinal surgery: what does an obstetrician need to know? *Archives of Gynecology and Obstetrics*. 2006;274(6):373–375. doi: 10.1007/s00404-006-0137-z 16547684

[24] Villevieille T, Mercier FJ, Benhamou D. Is obstetric epidural anaesthesia technically possible after spinal surgery and does it work? *Annales Francaises d’Anesthesie et de Reanimation*. 2003;22(2):91–95. S0750765802008572 [pii] doi: 10.1016/s0750-7658(02)00857-2 12706761

[25] Daley MD, Rolbin SH, Hew EM, Morningstar BA, Stewart JA. Epidural anesthesia for obstetrics after spinal surgery. *Regional anesthesia*. 1990;15(6):280–284. 2291882

