# Supplementary material for: Pregnancy and delivery after spine fracture or surgery: A nationwide population-based register study in Finland
Source: PLoS One. 2022 Aug 5;17(8):e0272579. doi: 10.1371/journal.pone.0272579 (PMC9355215; doi:10.1371/journal.pone.0272579)
Supplement: S1 Table — (PDF) [file pone.0272579.s001.pdf]

Supplementary Table 1: Definitions for ICD-10-codes and NOMESCO classification codes for fracture-related and other major spine operations included in this study.

ICD-10 Code	Definition
S120	Fracture of first cervical vertebra
S121	Fracture of second cervical vertebra
S122	Fracture of third cervical vertebra
S127	Multiple fractures of cervical vertebra
S128	Fracture of other parts of neck
S129	Fracture of neck, unspecified
S220	Fracture of thoracic vertebra
S221	Multiple fractures of thoracic vertebra
S320	Fracture of lumbar vertebra
S321	Fracture of sacrum
NOMESCO classification of major surgical procedure related to spine fracture	
Procedure code	Definition
NAJ 10	Anterior reduction of fracture of cervical spine
NAJ 12	Posterior reduction of fracture of cervical spine
NAJ 20	Anterior reduction of fracture of thoracic spine
NAJ 22	Posterior reduction of fracture of thoracic spine
NAJ 30	Anterior reduction of fracture of lumbar spine
NAJ 32	Posterior reduction of fracture of lumbar spine
NOMESCO classification of fusion surgery	
Procedure code	Definition
NAG 40	Anterior fusion of cervical spine without fixation
NAG 41	Anterior fusion of cervical spine with fixation
NAG 42	Posterior fusion of cervical spine with or without fixation
NAG 50	Anterior fusion of thoracic spine without fixation
NAG 51	Anterior fusion of thoracic spine with fixation
NAG 52	Posterior or lateral fusion of thoracic spine with fixation, 2-3 vertebrae
NAG 53	Posterior or lateral fusion of thoracic spine with fixation, more than 3 vertebrae
NAG 57	Anterior and posterior fusion of thoracic spine
NAG 60	Anterior fusion of lumbar spine with fixation
NAG 61	Posterior fusion of lumbar spine without fixation
NAG 62	Posterior fusion of lumbar spine with fixation, 2-3 vertebrae
NAG 63	Posterior fusion of lumbar spine with fixation, more than 3 vertebrae
NAG 65	Anterior and posterior fusion of lumbar spine
NAG 66	Posterior interbody fusion of lumbar spine, 2 vertebrae
NAG 67	Posterior interbody fusion of lumbar spine, more than 2 vertebrae
