# Supplementary material for: Pregnancy and delivery after spine fracture or surgery: A nationwide population-based register study in Finland
Source: PLoS One. 2022 Aug 5;17(8):e0272579. doi: 10.1371/journal.pone.0272579 (PMC9355215; doi:10.1371/journal.pone.0272579)
Supplement: S2 Table — (PDF) [file pone.0272579.s002.pdf]

Supplementary table 2: Frequencies of the spine operations and the mean age of the women at the time of operation in these subgroups during the study period.

Code	Definition	n	%	Mean age (SD)
NAJ 10	Anterior reduction of fracture of cervical spine	132	1.3	29.8 (10.7)
NAJ 12	Posterior reduction of fracture of cervical spine	34	0.3	35.6 (10.8)
NAJ 20	Anterior reduction of fracture of thoracic spine	7	0.1	27.1 (12.6)
NAJ 22	Posterior reduction of fracture of thoracic spine	236	2.4	32.2 (10.7)
NAJ 30	Anterior reduction of fracture of lumbar spine	28	0.3	31.1 (11.1)
NAJ 32	Posterior reduction of fracture of lumbar spine	505	5.1	30.6 (10.4)
NAG 40	Anterior fusion of cervical spine without fixation	3593	36.2	42.6 (5.3)
NAG 41	Anterior fusion of cervical spine with fixation	621	6.3	41.5 (6.6)
NAG 42	Posterior fusion of cervical spine with or without fixation	130	1.3	39.3 (8.6)
NAG 50	Anterior fusion of thoracic spine without fixation	14	0.1	27.0 (10.5)
NAG 51	Anterior fusion of thoracic spine with fixation	20	0.2	27.1 (12.3)
NAG 52	Posterior or lateral fusion of thoracic spine with fixation, 2-3 vertebrae	32	0.3	40.3 (9.1)
NAG 53	Posterior or lateral fusion of thoracic spine with fixation, more than 3 vertebrae	757	7.6	19.1 (7.2)
NAG 57	Anterior and posterior fusion of thoracic spine	17	0.2	30.8 (14.5)
NAG 60	Anterior fusion of lumbar spine with fixation	183	1.8	33.9 (11.5)
NAG 61	Posterior fusion of lumbar spine without fixation	188	1.9	36.3 (11.3)
NAG 62	Posterior fusion of lumbar spine with fixation, 2-3 vertebrae	2011	20.3	40.3 (7.7)
NAG 63	Posterior fusion of lumbar spine with fixation, more than 3 vertebrae	384	3.9	36.4 (11.3)
NAG 65	Anterior and posterior fusion of lumbar spine	469	4.7	38.9 (9.0)
NAG 66	Posterior interbody fusion of lumbar spine, 2 vertebrae	516	5.2	40.4 (7.6)
NAG 67	Posterior interbody fusion of lumbar spine, more than 2 vertebrae	52	0.5	42.4 (6.2)
