# Supplementary figures and images for: Pregnancy and delivery after spine fracture or surgery: A nationwide population-based register study in Finland
Source: PLoS One. 2022 Aug 5;17(8):e0272579. doi: 10.1371/journal.pone.0272579 (PMC9355215; doi:10.1371/journal.pone.0272579)

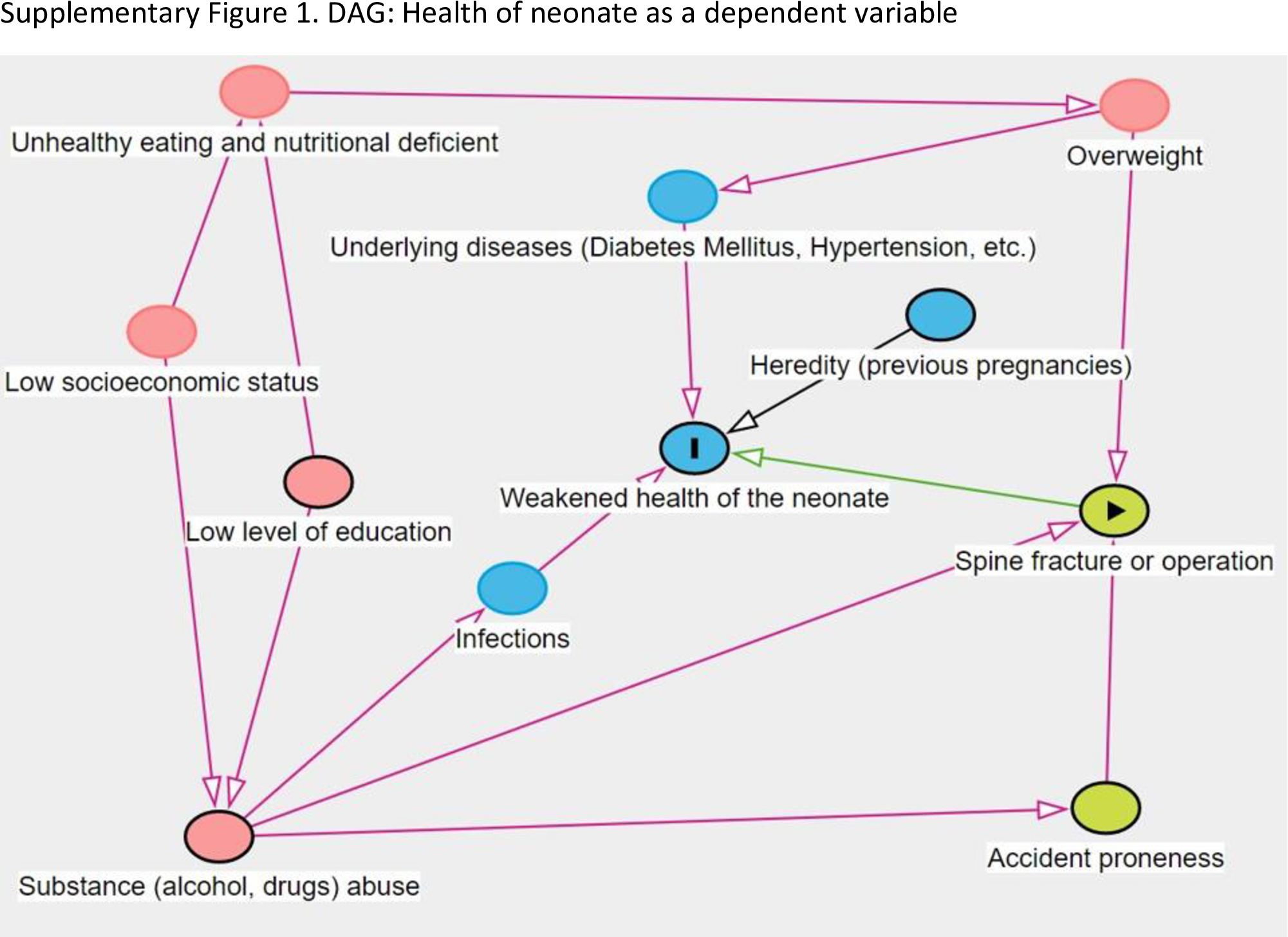

Supplement: S1 Fig — (TIF) [file pone.0272579.s003.tif]

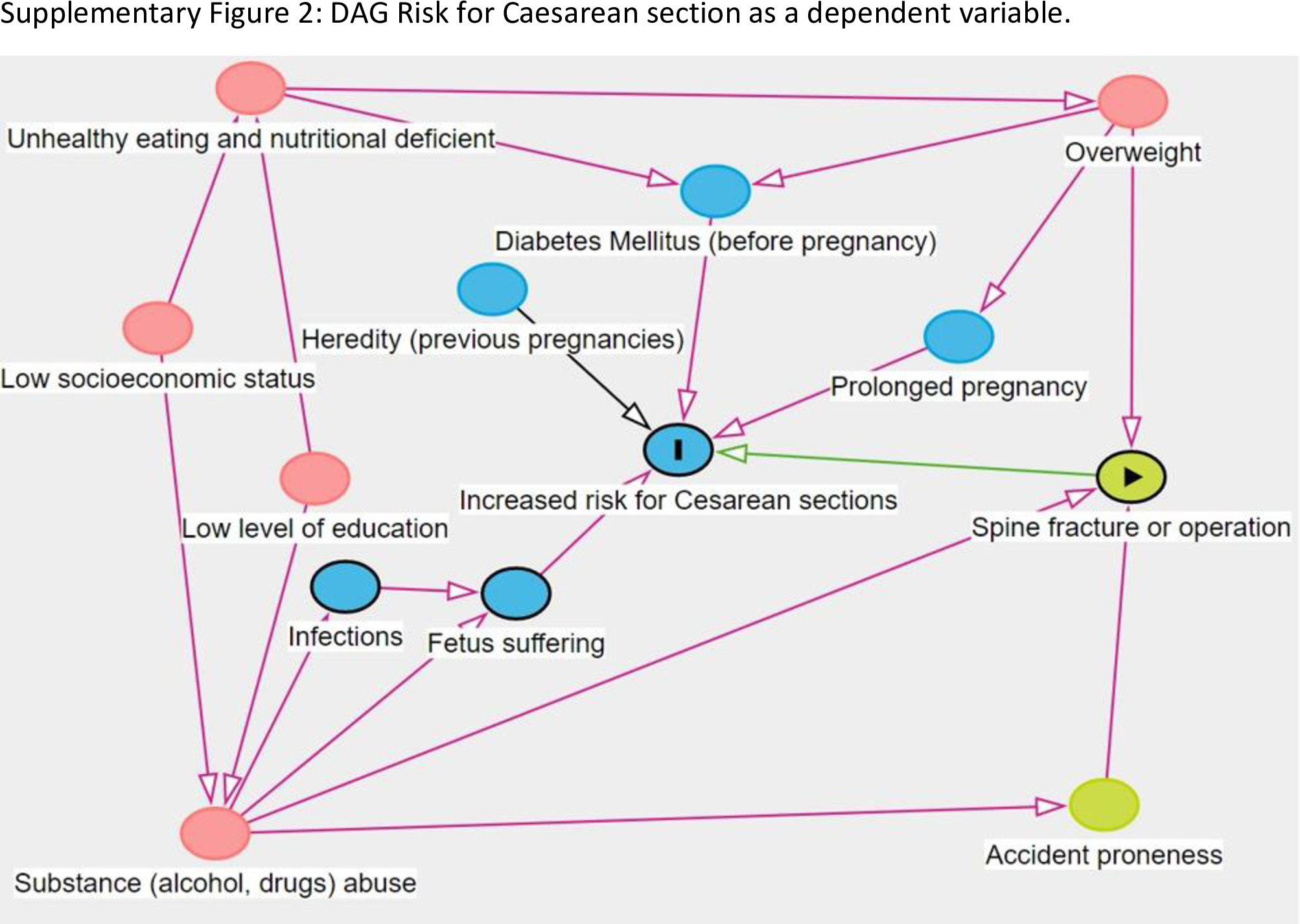

Supplement: S2 Fig — (TIF) [file pone.0272579.s004.tif]
